# Herpes Virus Reactivation in Astronauts During Spaceflight and Its Application on Earth

**DOI:** 10.3389/fmicb.2019.00016

**Published:** 2019-02-07

**Authors:** Bridgette V. Rooney, Brian E. Crucian, Duane L. Pierson, Mark L. Laudenslager, Satish K. Mehta

**Affiliations:** ^1^GeoControl Systems, Inc., Houston, TX, United States; ^2^NASA Johnson Space Center, Houston, TX, United States; ^3^Anschutz Medical Campus, University of Colorado Denver, Aurora, CO, United States; ^4^Jes Tech, KBR Wyle Laboratories Houston, Houston, TX, United States

**Keywords:** herpes, latency, viral reactivation, spaceflight, immunity

## Abstract

Latent herpes virus reactivation has been demonstrated in astronauts during shuttle (10–16 days) and International Space Station (≥180 days) flights. Following reactivation, viruses are shed in the body fluids of astronauts. Typically, shedding of viral DNA is asymptomatic in astronauts regardless of mission duration; however, in some cases, live/infectious virus was recovered by tissue culture that was associated with atopic-dermatitis or skin lesions during and after spaceflight. Hypothalamic-pituitary-adrenal (HPA) and sympathetic-adrenal-medullary (SAM) axes activation during spaceflight occurs as indicated by increased levels of stress hormones including cortisol, dehydroepiandrosterone, epinephrine, and norepinephrine. These changes, along with a decreased cell mediated immunity, contribute to the reactivation of latent herpes viruses in astronauts. Currently, 47/89 (53%) astronauts from shuttle-flights and 14/23 (61%) astronauts from ISS missions shed one or more herpes viruses in saliva/urine samples. Astronauts shed Epstein–Barr virus (EBV), varicella-zoster virus (VZV), and herpes-simplex-1 (HSV-1) in saliva and cytomegalovirus (CMV) in urine. Larger quantities and increased frequencies for these viruses were found during spaceflight as compared to before or after flight samples and their matched healthy controls. The shedding did not abate during the longer ISS missions, but rather increased in frequency and amplitude. These findings coincided with the immune system dysregulation observed in astronauts from shuttle and ISS missions. VZV shedding increased from 41% in space shuttle to 65% in ISS missions, EBV increased 82 to 96%, and CMV increased 47 to 61%. In addition, VZV/CMV shed ≤30 days after ISS in contrast to shuttle where VZV/CMV shed up to 5 and 3 days after flight respectively. Continued shedding of infectious-virus post-flight may pose a potential risk for crew who may encounter newborn infants, sero-negative adults or any immunocompromised individuals on Earth. Therefore, developing spaceflight countermeasures to prevent viral reactivation is essential. Our spaceflight-developed technologies for saliva collection/rapid viral detection have been extended to include clinical applications including zoster patients, chicken pox, post-herpetic neuralgia, multiple sclerosis, and various neurological disorders. These protocols are employed in various clinics and hospitals including the CDC and Columbia University in New York, as well as overseas in Switzerland and Israel.

## Introduction

### Herpes Virus

Herpes viruses have co-evolved with humans for millennia and subsequently employ sophisticated strategies to evade the host immune response. Consequently, after primary infection, they persist lifelong in a latent or dormant phase, and are generally asymptomatic in immunocompetent individuals. However, they may reactivate during periods of increased stress, isolation, and during times of immune challenge. Eight major herpes viruses parasitize humans with worldwide infection rates of 70–95%. Four of the eight are shed in the body fluids of NASA astronauts during both short and long duration spaceflight. Though viral load (virus detected in the body fluids) can be high, these astronauts often have no clinical symptoms associated with reactivation (Mehta et al., 2004). Post-reactivation, replication of the virus may also be enhanced which could account for the significant increase in viral shedding during spaceflight. Yet, there have been a few cases where the reactivation culminated in commensurate atopic dermatitis and/or viral lesions (Crucian B. E. et al., 2016).

### Astronaut Stress/Exposures

Exposure of astronauts, during both short and long duration spaceflight, to non-terrestrial hazards such as variable gravitational forces including acceleration/deceleration, cosmic radiation, and microgravity result in a unique set of stressors that contribute to the dysregulation of the immune and endocrine systems (Crucian and Sams, 2009; Crucian et al., 2013). In addition, they also endure some common stressors including but not limited to social separation, confinement, sleep deprivation, circadian rhythm disruption, and anxiety. There is increasing evidence to suggest that these spaceflight-associated stressors chronically amplify the release of stress hormones, which negatively affects the immune system, especially the adaptive immune system facilitating latent herpes virus reactivation during and after spaceflight. Increased levels of salivary, plasma and urinary stress hormones such as cortisol and catecholamines commonly accompany spaceflight (Stowe et al., 2001a).

### Altered Immunity

Maintenance of viral latency requires a vigorous and vigilant immune system, highly dependent upon competent cytotoxic T-cells, and any changes in immune status tend to promote viral reactivation. This is evident in both terrestrial space-analog studies (Crucian B. et al., 2014) and spaceflight studies (Stowe et al., 2001b, 2011; Mehta et al., 2014, 2017). The alterations in immune status for terrestrial analog studies are minor and coincide with mild viral reactivation. Spaceflight studies illustrate major immune dysregulation and functional changes in conjunction with significant viral reactivation, regardless of mission duration. In fact, substantial changes in cell-mediated immunity exist in most astronauts that reactivated one or more herpes viruses (Mehta et al., 2014; Crucian et al., 2015). This was also highlighted by Glaser et al. (1993), who previously showed an association of EBV reactivation and diminished cell-mediated immunity.

**FIGURE 1:**
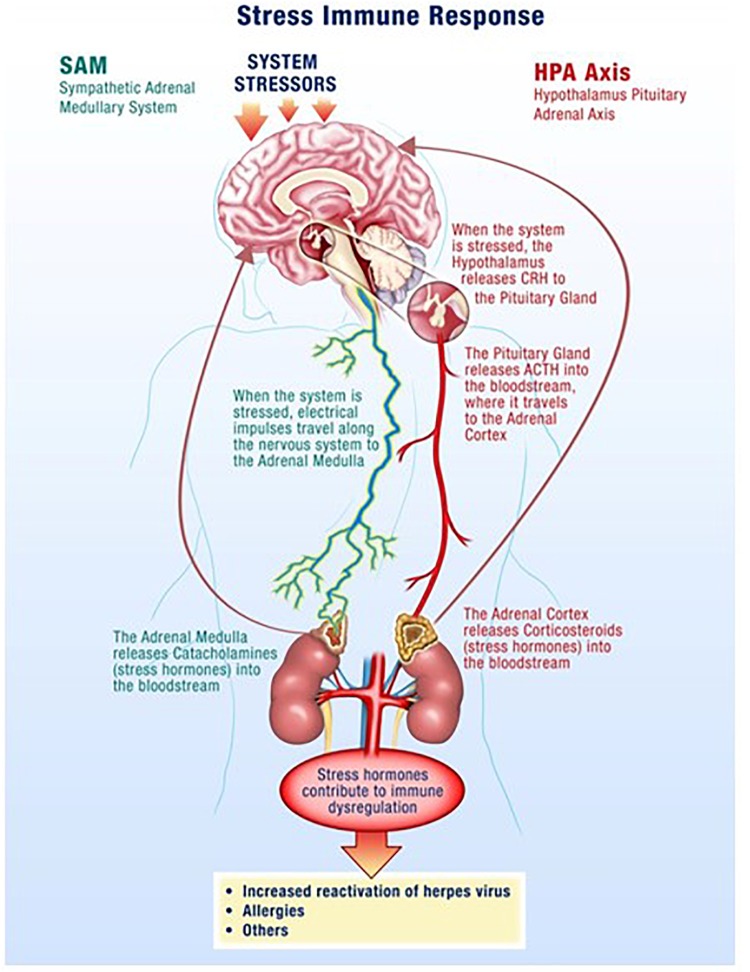
Spaceflight is a stressful environment with various stressors acting through the hypothalamus-pituitary-adrenal (HPA)-axis and the sympathetic-adrenal-medullary (SAM)-axis. Increases in stress hormones, such as cortisol from the adrenal glands, result in reductions in cellular immunity which facilitates opportunistic viral reactivation.

The hypothalamus-pituitary-adrenal (HPA) axis along with the sympathetic-adrenal-medullary (SAM) axis partially mediate the stress response where glucocorticoids and catecholamines are secreted in proportionate concentrations relative to the stress stimulus (Figure 1) (Padgett and Glaser, 2003; Webster and Glaser, 2008; Goldstein, 2010). Though acute responses to stress can be positive, long duration or chronically high levels of stress hormones can negatively affect the regulation of the immune system and its individual components (Crucian B. E. et al., 2014). Changes in a variety of immune cells, both in form (phenotype) and function (killing capacity), result in decreased cell-mediated immunity, which facilitates opportunistic reactivation of latent viruses (Crucian et al., 2015; Bigley et al., 2017).

#### Stress Hormones/Cytokines

**FIGURE 2:**
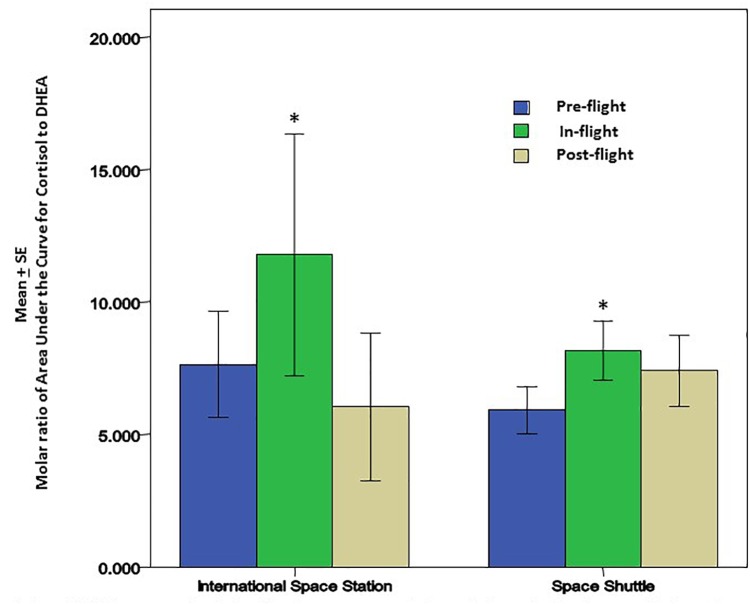
Cortisol and DHEA were analyzed in saliva from astronauts before, during and after the space flights using a commercially available ELISA assays (Salimetrics, LLC, State College, PA, United States). There was a significant increase in the molar ratio of cortisol to DHEA during the flight phase for both Space Shuttle (*N* = 17) or ISS (*N* = 10). The increase in this ratio may be associated with lower cellular immunity and innate immunity; potentially contributing to greater inflammatory cytokines that may affect bone remodeling and bone growth. ^∗^ Indicates significance when comparing flight against pre-flight and post-flight. *p* < 0.01.

Cortisol and dehydroepiandrosterone (DHEA) are glucocorticoid steroid hormones released by the adrenal glands in response to stress. Cortisol is anti-inflammatory and immunosuppressive, but DHEA is an important antagonist to cortisol. For that reason, the molar ratio of cortisol to DHEA [C]/[D] is an important indicator of immune regulation. In recent flight studies, the regular diurnal release of these hormones was tracked in saliva samples to evaluate any changes/trends occurring through the various phases of flight; launch/pre-flight, flight, and return (Mehta et al., 2014). Salivary cortisol was present in significantly higher concentrations in samples taken before and during flight. Salivary DHEA followed its normal daily decline kinetics in the samples taken before, during and after flight, but has been found to have significantly lower waking concentrations during the flight phase in comparison to samples taken before and after flight. Altogether, diurnal patterns of salivary cortisol were significantly higher during flight while DHEA was significantly lower. The cortisol area under the curve relative to ground (AUCg) did not change significantly during flight relative to baseline whereas DHEA AUCg significantly declined during flight relative to baseline. Ultimately, this results in an increased [C]/[D] molar ratio during spaceflight (Figure 2) which potentially indicates immune challenge, and has been linked to immune modulation (Christeff et al., 1997), including the increased inflammatory cytokine response and the TH2 shift observed in earlier spaceflight studies (Mehta et al., 2013a; Crucian B. E. et al., 2014).

Cytokines are small cell-signaling proteins that play a crucial role in the modulation of the human immune response. They can facilitate both pro- and anti-inflammatory immune states and are generally analyzed in the categories of inflammatory cytokines (IL-1α, IL-1β, TNFα, IL-6, IL-8), lymphoid growth factors (IL-2, IL-7, IL-15), Th1/17 cytokines (IFNγ, IL-12, IL-17), Th2 cytokines (IL-4, IL-5, IL-10, IL-13), myeloid growth factors (G-CSF, GM-CSF), and chemokines (eotaxin, MCP-1, M1P1α, IP-10). Recent flight studies (Mehta et al., 2013a; Crucian B. E. et al., 2014; Crucian et al., 2015) have shown that astronauts displayed significant increases in the pro-inflammatory plasma cytokines IL-1α, IL-6, IL-8, IFNγ, IL-4, eotaxin, and IP-10 in samples taken 10 days before launch (L-10), in comparison to their baseline samples taken 180 days before launch (L-180). The increase of IL-6, IL-8, IL-4, eotaxin, and IP-10 is also evident immediately upon return to Earth at landing, designated as R+0. The Th2 cytokine IL-4 was the most sensitive/responsive to the phases of flight with 35- and 21-fold increases from baseline values at L-10 and R+0, respectively.

When analyzing plasma cytokine levels in the context of virus shedding, there seems to be a connection between astronauts who shed virus and significantly elevated levels of cytokines (IL-1α, IL-6, IL-8, IFNγ, IL-12p70, IL-4, IL-10, IL-13, eotaxin, and IP-10) (Mehta et al., 2013a). Lymphoid and myeloid growth factors are also elevated in virus shedding astronauts, by about twofold. As mentioned earlier, the Th2 cytokine IL-4 shows the largest fold increases through launch and return flight phases, and this is evident again when restricting the analysis to only viral-shedding astronauts at the return time point R+0. For these astronauts, the single largest plasma cytokine increases were IL-4 (21-fold increase) and IL- 6 (33-fold increase). This indicates a dynamic shift from a Th1 antiviral immune state to a Th2 antibacterial/antifungal immune state. Further emphasizing the Th1-Th2 shift is an analysis of the ratio of IFNγ: IL-4. The results from some of the most recent flight studies suggest a significant decrease in the IFNγ: IL-4 ratio for shedders compared to astronauts who did not shed any viruses during their duty rotation (Mehta et al., 2013a; Crucian B. E. et al., 2014).

#### Viral Specific T-Cell and NK-Cell Function

Alterations in the aforementioned cytokines play a critical role in the fate of many important leukocyte populations. The cytokine profile changes, acting either independently or in conjunction with microgravity, generate a variety of immune vulnerabilities by significantly changing the numbers, proportions, and functions of leukocytes. Monocyte (Kaur et al., 2005), granulocyte (Stowe et al., 1999), and lymphocyte functions (Crucian et al., 2015; Bigley et al., 2017) are diminished, critically reducing the effectiveness of the immune response to pathogens, as well as its capacity to prevent viral reactivation. T-Cells and NK-Cells in particular, which function to attack and destroy viruses/virally infected cells, are substantially debilitated during spaceflight. Flight studies focusing on T-Cell function have elucidated that both CD4+ and CD8+ T-Cells taken from astronauts during the flight phase respond ineffectively against a variety of stimuli. Under normal circumstances, these same stimuli would have elicited a more profound response by the T-Cells. The weakened response can last the duration of the flight phase (Crucian et al., 2015). Additional flight studies focusing on the function of NK-Cells have shown decrements in cytotoxicity due to decreased production of the enzymes perforin and granzyme B (Bigley et al., 2017). Without these enzymes, NK-Cells are rendered ineffective against the target cell/pathogen and this impairment may last up to 60 days post-flight. In both cases, reductions in T-Cell and NK-Cell function lead to the inability of the immune system to suppress/sequester/eliminate opportunistic viral reactivation.

### Viral Latency

As stated earlier, herpes viruses share a long-term co-evolutionary history with humankind. This promotes a relatively benign life-long persistence of the virus within the host. In healthy individuals with robust immune surveillance, viral activity can occur in the absence of clinical symptoms (Grinde, 2013). Injury to the host is antithetical to viral survival. Viral persistence in the host is aided by viral strategies for latency. Latency is a well-orchestrated series of concomitant events that allow for viral genome maintenance, while actively repressing lytic (replicative) gene expression and promoting latent gene expression. A hallmark of viral latency is that infectious viral progeny are not produced, so the surrounding cells remain uninfected or naïve. Viral latency is the culmination of a handful of factors; infection of cell types permissive to latency, viral promotion of infected cell survival, and the general evasion of the host immune response. Cellular tropism is dictated by cell surface receptor expression, as well as intracellular conditions permissive to viral activity, and is very specific to the individual herpes viruses. HSV and VZV infect neurons in ganglia, while EBV and CMV preferentially infect the cells of the immune system, B cells and myeloid progenitor cells, respectively. Other cell types can be infected by the viruses, but the aforementioned cell types serve as the greater viral latent reservoirs. The promotion of infected cell survival is the product of viral manipulation of host cell machinery. For example, the manipulation of Bcl-2 family proteins promotes survival of CMV infected monocytes (Collins-McMillen et al., 2015). Not only can herpes viruses manipulate the cells they infect, but they can also affect the host immune response. Interestingly, latent viruses are still very genetically active even in the absence of replication. There is emerging evidence of significant miRNA activity during latency that can act to override lytic transcription, as well as to alter the cell secretome. Though the role of miRNA are yet to be fully teased out, they seem to facilitate the transcription of proteins that mimic host cytokines and chemokines which ultimately inhibit host anti-viral activity. For the many nuances of viral latency, specific to each virus, the reader is directed to the following review articles (Eshleman et al., 2011; Nicoll et al., 2012; Kempkes and Robertson, 2015; Wills et al., 2015).

### Viral Reactivation

**FIGURE 3:**
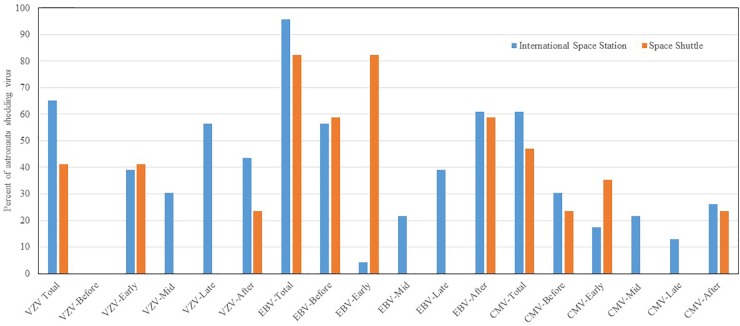
Percent distribution of astronauts shedding VZV. EBV and CMV before, during the mission at time point early-mission, mid-mission, late-mission, and after either short and long duration space flights. Saliva and urine samples were collected from 112 astronauts (89 short duration and 23 long duration) before, during, and after the spaceflight. Saliva was analyzed for Epstein–Barr virus (EBV) and varicella-zoster virus (VZV), and urine was analyzed for Cytomegalovirus CMV by real time PCR assay using Taqman 7900 (Thermofisher, Inc.). The shedding of EBV. VZV and CMV DNA in body fluids is significantly higher during spaceflight as compared to pre-flight, post-flight, and the control *p* < 0.01 (Mehta et al., 2014, 2017). However, when comparing these shedding patterns between space shuttle and ISS missions, the differences were not significant.

Reactivation and shedding of latent herpes viruses has been reported in astronauts during space shuttle, Russian Soyuz and International Space Station missions (Pierson et al., 2005, 2007; Mehta and Pierson, 2007; Mehta et al., 2014, 2017). Virus reactivation has also been observed in ground-based models of spaceflight including Antarctica, undersea habitat, artificial gravity and bed rest studies, though not to the extent seen during spaceflight studies. So far, 47 out of 89 (53%) astronauts from short duration space shuttle flights, and 14 out of 23 (61%) from long duration ISS spaceflight missions shed at least one or more herpes viruses in their saliva or urine samples. Significant reactivations of EBV, CMV, and VZV occurred during flight phase and the magnitude and frequency of viral shedding during spaceflight directly correlates with duration of spaceflight. VZV shedding increased from 41% in space shuttle to 65% in ISS missions, EBV increased from 82 to 96%, and CMV increased from 47 to 61%. In addition, VZV and CMV shed up to a month post-long duration flight. Percent distribution of these viruses during shuttle and ISS missions is depicted in Figure 3. These viruses often reactivate in concert with one another, but they may also reactivate independently of the other viruses. Reactivation of latent viruses during long-duration spaceflight could increase risk for adverse medical events during exploration-class deep-space missions (Crucian and Sams, 2009). Taken altogether, and to our knowledge, there have been six incidences of astronauts with complaints of symptoms related to herpes viral reactivation (Crucian B. et al., 2016). VZV is an important health risk to crewmembers (several have experienced shingles during flight). Furthermore, CMV can be immuno-suppressive and may play a role in the well-documented immune dysfunction observed in crewmembers.

#### Epstein–Barr Virus

Epstein–Barr virus (EBV) is responsible for infectious mononucleosis and is associated with several malignancies (Niedobitek et al., 2001; Thompson and Kurzrock, 2004; Bravender, 2010). It is a highly infectious DNA virus transmitted by aerosolized micro-droplets and by direct contact with saliva. It has a 95% infection rate among adults worldwide, which makes it an ideal target for investigation among a limited and unique astronaut population. EBV preferentially infects B-lymphocytes and these cells serve as a latent virus reservoir. Early flight studies from the shuttle missions were the first to demonstrate that EBV DNA was shed in astronaut saliva samples taken before, during, and after space flight (Stowe et al., 2001b; Pierson et al., 2005; Mehta et al., 2014). These studies highlighted a 10-fold increase in viral load during the flight phase in comparison to samples taken before or after flight. Additionally, EBV copies shed during space flight seemed to increase as a function of time in space, and as a result of diminished cell immunity (Mehta et al., 2000a). These early findings have been repeatedly corroborated in longer duration ISS missions (Stowe et al., 2011; Mehta et al., 2017). Altogether, flight studies have illustrated that approximately 90% of astronauts, regardless of mission duration, shed EBV during spaceflight.

#### Varicella Zoster Virus

Varicella Zoster virus (VZV) is highly communicable and responsible for both chicken pox (primary infection) and shingles (secondary infection). The virus is transmitted via saliva and can be aerosolized by sneezing and coughing. After primary infection, VZV becomes latent in various nerve ganglia (Reichelt et al., 2008) (cranial, dorsal root, autonomic) along the entire length of the neuroaxis, and reactivation often results in characteristic skin lesions that range from aggravating to painful. Though reactivation of VZV is evident from flight studies where viral DNA was found in saliva of astronauts from both shuttle and ISS missions, astronauts do not often develop symptoms or rash (Mehta et al., 2004; Cohrs et al., 2008). Additionally, we have found that saliva samples taken 2–6 days following landing were infectious by culturing that saliva with human fetal lung (HFL) cells. Infectious VZV was present and confirmed by visual inspection of the culture where viral plaques were obvious, as well as by antibody staining and real-time PCR DNA analysis. This poses a risk to the welfare of both astronauts and their seronegative contacts back on Earth, as VZV viral load also increases with time in space and is present in saliva of about 60% of astronauts from combined shuttle and ISS missions.

#### Herpes Simplex Virus-1

Herpes Simplex virus-1 is highly prevalent and communicable and persists as a latent virus lifelong. Generally referred to as oral herpes, reactivation can be either asymptomatic or lead to lesions/rash anywhere on the body. Incidences of HSV-1 reactivation are very low with only 8% of astronaut saliva samples test positive for viral DNA, though recently, an astronaut suffering persistent dermatitis during a long duration spaceflight, >180 day, was positive for HSV-1 viral DNA in saliva and lesion samples. The saliva containing virus was infectious, as evidenced by a culture of the saliva atop HFL cells, where visual disruption of HFL cells was apparent at 3 days post-infection with saliva, with viral load verified/quantified by real-time PCR.

#### Cytomegalovirus

Cytomegalovirus is the only beta-herpes virus known to reactivate in astronauts. It is typically acquired asymptomatically during childhood and has a worldwide prevalence of 75–90%. Though it remains asymptomatic in immunocompetent people, it may reactivate in individuals whose immune systems are either immature or immunocompromised causing multiple diseases such as encephalitis, gastroenteritis, pneumonia, and chorioretinitis (Seitz, 2010). Moreover, several studies have suggested that CMV infection is immunosuppressive because it directly infects leukocytes as well as hematopoietic cells (Varani and Landini, 2011; O’Connor and Murphy, 2012; Stevenson et al., 2014). Additionally, CMV has been uniquely linked to early immune senescence (Pawelec, 2014; Sansoni et al., 2014). However, a study was able to illustrate a benefit of CMV infection specifically to young adults (20–30 years old) regarding increased antibody response to the influenza vaccine (Furman et al., 2015). Spaceflight studies have shown that 27% of the astronauts from short-term space missions shed CMV DNA in either pre- or post-flight urine samples, and that anti-CMV IgG antibody titers increased significantly for all shedders from each time point compared to their baseline values (Mehta et al., 2000b). In long duration spaceflight, 61% of astronauts shed CMV DNA in their urine during and after spaceflight in stark contrast to the absence of CMV DNA in urine samples taken 180 days before flight. These findings demonstrate that CMV reactivation occurs in astronauts regardless of mission duration, and this may pose additional threats to the health of crewmembers during longer-duration missions (Mehta et al., 2014, 2017).

### Rapid Detection of Virus for Application in Patient Populations

The most obvious signs of VZV reactivation are the vesicular rash and the pain associated with zoster, however even in the absence of rash, the virus is active and can spread to the retina causing blindness, to the spinal cord causing paralysis and incontinence, and to cerebral arteries resulting in stroke (Kleinschmidt-DeMasters and Gilden, 2001; Orme et al., 2007). Associating VZV with a disease asymptomatically can be challenging. For example, when stroke occurs in the elderly, especially many months following zoster, the association with VZV reactivation requires cerebral spinal fluid (CSF) analysis for VZV antibodies (Nagel et al., 2007). Likewise, detection of asymptomatic VZV reactivation, which often is only seen as an increase in antibody titer against VZV (but may also result in virus transmission), is difficult to detect. In such instances, virological verification of VZV disease has relied on the detection of VZV DNA or anti-VZV IgG antibodies in CSF or, less often, the presence of VZV DNA in blood mononuclear cells or anti-VZV IgM antibodies in serum.

**FIGURE 4:**
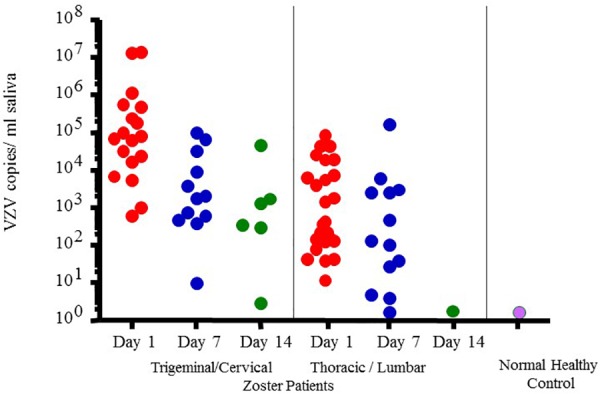
Varicella-zoster virus (VZV) copy number per mL of saliva in Fifty-four zoster patients treated with valacyclovir and 14 healthy subjects. On treatment days 1, 7-, and 14 later, pain was scored (data not given here) and saliva examined for VZV-DNA by real time PCR. Patients were divided into two groups based upon the infected dermatome, Trigeminal Cervical and Thoracic/Lumbar. VZV-DNA was found in every patient the day treatment was started and disappeared in 82% with the treatment. Analysis of human saliva has potential usefulness in diagnosing neurological disease produced by VZV without rash. When comparing patient shedding against normal healthy controls, it was significantly higher, *p* < 0.01 (Mehta et al., 2008).

However, VZV DNA has been detected in the saliva samples from patients with acute zoster (Mehta et al., 2008), zoster sine herpete (Hato et al., 2000), chickenpox (Mehta et al., 2013b) and post-herpetic neuralgia (PHN) (Nagel et al., 2011) even before the rash appears, which now makes diagnosis less invasive and less time consuming. In fact, a rapid and sensitive virus detection method has been developed and used to detect virus in saliva samples taken from asymptomatic patients with neurologic and other VZV related disease (Mehta et al., 2013b). Figure 4 illustrates VZV copy numbers in saliva from shingles patients before anti-viral treatment. For this method, the saliva is collected by passive drool or by way of a synthetic swab and then processed for DNA within an hour from sample collection. The results from a few studies using this technique have shown that VZV DNA is present in 100% of patients tested before antiviral treatment and is exclusively in the cell pelleted fraction of saliva. These studies further showed that VZV, isolated from zoster patient saliva, was primarily associated with the epithelial cell membrane but could also be inside the cell. Epithelial cells with VZV continued to be present in the saliva of a single zoster patient up to 10 months after recovery. These kinds of studies are ongoing and our spaceflight-developed technology for rapid viral detection continues to be used locally and around the world for patients with zoster (Mehta et al., 2013b), chicken pox (Mehta et al., 2008), PHN (Nagel et al., 2011), multiple sclerosis (Ricklin et al., 2013), and various other neurological disorders (Gilden et al., 2010; Pollak et al., 2015).

## Conclusion

Reactivation of latent viruses is a powerful biomarker of immune status for astronauts deployed to space. There are multiple factors that influence reactivation including increases in glucocorticoid/catecholamine secretion, cytokine profile shifts, and decreased function in the major leukocyte and lymphocyte subsets designed to suppress and eliminate viruses/virally infected cells. Viral reactivation is evident through the shedding of viral DNA in the body fluids of astronauts, and the viral load only increases with more time in space. Additionally, more than one virus generally reactivates at a time, potentially compounding the physiological ramifications of uncontrolled viral reactivation to not only rashes, but also severe target organ failures, and permanent vision and hearing loss. The occupational hazards for astronauts are profound, but research into the causes and mechanics of viral reactivation not only benefit the astronaut but also the general patient population. As our understanding of viral reactivation widens, we are better able to develop and implement effective countermeasures for our astronaut professionals, as well as targeted treatment regimens for immunocompromised individuals suffering the consequences of viral reactivation. As a result, this research has tremendous clinical relevance.

Ultimately, the information gleaned from these space studies will shape the way we prepare for and design exploration-class missions, beyond the moon and mars, where reactivation of latent viruses could result in increased risk for wide-ranging adverse medical events. Partial-gravity environments, e.g., on Mars, might be sufficient to curtail serious viral reactivation, but this needs to be addressed in future research. In the interim, because astronaut saliva contains increasingly significant viral DNA, during and after spaceflight that can be infectious, we recommend prophylactics (vaccines), where available, to the astronauts before they go into space.

## Author Contributions

SM designed and executed the study, collected and processed the samples from astronauts, analyzed the data, and wrote the manuscript. DP designed the study and wrote the manuscript. BC executed the study and wrote the manuscript. BR wrote the manuscript. ML contributed to the measurement of salivary cortisol and DHEA processing and manuscript preparation.

## Conflict of Interest Statement

BR was employed by GeoControl Systems Incorporated and SM was employed by Jes Tech, KBR Wyle Laboratories. The remaining authors declare that the research was conducted in the absence of any commercial or financial relationships that could be construed as a potential conflict of interest.
